# Hospital laboratory reporting may be a barrier to detection of ‘microsize’ myocardial infarction in the US: an observational study

**DOI:** 10.1186/1472-6963-13-162

**Published:** 2013-05-01

**Authors:** Monika M Safford, Gaurav Parmar, Codrin S Barasch, Jewell H Halanych, Stephen P Glasser, David C Goff, Ronald J Prineas, Todd M Brown

**Affiliations:** 1 Division of Preventive Medicine, University of Alabama at Birmingham School of Medicine, Medical Towers 621, 1717 11th Avenue South, Birmingham, AL 35294-4410, USA; 2 Colorado School of Public Health, Building 500, 3rd Floor, Suite 300, Anschutz Medical Campus, Aurora, CO 80045, USA; 3 Department of Epidemiology & Prevention, Wake Forest University School of Medicine, Medical Center Boulevard, Winston-Salem, NC 27157-1063, USA; 4 Division of Cardiovascular Disease, University of Alabama at Birmingham School of Medicine, 701 19th Street South, Birmingham, AL 35294-0007, USA

**Keywords:** Acute coronary syndrome, Troponin, Quality control

## Abstract

**Background:**

International guidelines recommend that the decision threshold for troponin should be the 99^th^ percentile of a normal population, or, if the laboratory assay is not sufficiently precise at this low level, the level at which the assay achieves a 10% or better coefficient of variation (CV). Our objectives were to examine US hospital laboratory troponin reports to determine whether either the 99^th^ percentile or the 10% CV level were clearly indicated, and whether nonconcordance with these guidelines was a potential barrier to detecting clinically important microscopic or ‘microsize’ myocardial infarctions (MIs). To confirm past reports of the clinical importance of microsize MIs, we also contrasted in-hospital, 28-day and 1-year mortality among those with microsize and nonmicrosize MI.

**Methods:**

In the REasons for Geographic And Racial Differences in Stroke national prospective cohort study (n=30,239), 1029 participants were hospitalized for acute coronary syndrome (ACS) between 2003–2009. For each case, we recorded all thresholds of abnormal troponin on the laboratory report and whether the 99^th^ percentile or 10% CV value were clearly identified. All cases were expert adjudicated for presence of MI. Peak troponin values were used to classify MIs as microsize MI (< five times the lowest listed upper limit of normal) and nonmicrosize MI.

**Results:**

Participants were hospitalized at 649 acute care US hospitals, only 2% of whose lab reports clearly identified the 99^th^ percentile or the 10% CV level; 52% of reports indicated an indeterminate range, a practice that is no longer recommended. There were 183 microsize MIs and 353 nonmicrosize MIs. In-hospital mortality tended to be lower in the microsize than in the nonmicrosize MI group (1.1 vs. 3.6%, p = 0.09), but 28-day and 1-year mortality were similar (2.5% vs. 2.7% [p = 0.93] and 5.2% vs. 4.3% [p = 0.64], respectively).

**Conclusions:**

Current practices in many US hospitals created barriers to the clinical recognition of microsize MI, which was common and clinically important in our study. Improved hospital troponin reporting is warranted.

## Background

Guidelines for defining myocardial infarction (MI) have long recommended that events be classified using a combination of the clinical presentation, electrocardiogram (ECG) or cardiac imaging findings, and biomarkers [1-4]. Troponin is the preferred biomarker due to its high specificity for cardiac muscle [1-4]. The risk for mortality and cardiovascular events is increased with even modest troponin elevations [5-9]; therefore, for several years, American and European cardiology societies, the National Heart, Lung and Blood Institute, the Centers for Disease Control and Prevention, and others have recommended defining abnormal troponin as any value above the 99^th^ percentile for healthy individuals [1-4,10-14], culminating in the publication of the Universal Definition of MI in 2007 [1-3].

A problem acknowledged by the Universal Definition and other preceding guidelines is that some assays may not be reliable at levels as low as the 99^th^ percentile. Troponin levels in normal reference populations are very close to zero, and recent advances in biomarker assays permit detection of levels as low as 0.006 μg/L [15]. However, precision at very low levels may not be acceptable [15]. Therefore, until assays achieve greater precision at very low levels and become more standardized, experts have recommended defining the threshold for abnormal troponin as either the 99^th^ percentile of a normal reference population or the level at which the assay achieves acceptable precision, defined by a coefficient of variation (CV) of 10% or better [1-4,10-14]. All values of troponin above this threshold are recommended to be considered myocardial necrosis, which, together with a characteristic rising and/or falling pattern, is used in clinical decision-making to classify an event as an MI.

In order to apply this recommendation, the 99^th^ percentile or 10% CV level must be clearly identified on hospital laboratory troponin reports and labeled as the decision threshold. However, many hospitals have for years reported an “indeterminate range,” which was useful in interpreting troponin relative to creatine kinase (CK)-MB fractions when troponin was first introduced. The upper limit of this indeterminate range was the World Health Organization (WHO) cut-point corresponding to abnormal CK-MB, and many reports continue to provide interpretive guidelines recommending that troponin values above this WHO cut-point represent a high likelihood of MI. Clinicians interpreting such reports today may be challenged to identify the more current, lower guideline-recommended decision threshold. Furthermore, as ST elevation MI is declining while non-ST elevation MI remains common [16,17], troponin levels are increasingly the deciding factor in determining whether acute coronary syndrome (ACS) is classified as an MI. In this context, the troponin laboratory report is critical for appropriately classifying and risk stratifying MI events. However, guideline concordance in hospital laboratory troponin reports has to our knowledge not been examined.

We studied US hospital laboratory troponin practices in the REasons for Geographic And Racial Differences in Stroke (REGARDS) study, a large national prospective cohort study. We also examined mortality risks associated with very low, or ‘microsize,’ troponin peak MIs relative to MIs with higher peak troponin levels to both confirm the risks that have been described in other populations and to highlight the potential clinical relevance of hospital troponin reporting practices.

## Methods

The REGARDS study is prospectively following 30,239 individuals for cardiovascular events and mortality to better understand regional and racial influences on stroke and MI mortality. Details of the study are described elsewhere [18]. Briefly, recruitment was conducted from 2003–2007 using commercially available lists and a combination of mail and telephone contact to recruit English-speaking, community-dwelling adults aged 45 and older living in the 48 contiguous US. Baseline data collection included telephone surveys and in-home exams, and living participants are telephoned every six months and asked if they were hospitalized with subsequent medical records retrieval. Deaths were detected from reaching next of kin at a scheduled follow-up, online sources (e.g., Social Security Death Index), or the National Death Index. Medical records were reviewed by a team of experts, who adjudicated MI events using a standardized approach modeled on major epidemiologic studies [4]. The study protocol was reviewed and approved by the University of Alabama Institutional Review Board, and all participants provided informed consent.

We included the first hospitalization for ACS for each participant occurring between 2003 and 2009. ACS was defined as an urgent presentation of signs and symptoms suggestive of acute coronary ischemia resulting in hospitalization. For each case, we examined the hospital laboratory troponin report for documentation of the 99^th^ percentile for healthy adults or the 10% CV level for the assay utilized and whether either was clearly identified as the decision threshold for abnormal troponin. We also examined whether an indeterminate range was provided, a practice that is no longer recommended [1].

Expert adjudicators classified cases as ACS without MI or ACS with MI following approaches used in most large epidemiology studies [4]. For MI, medical records were examined for the presence of signs or symptoms suggestive of ischemia; a rising and/or falling pattern in cardiac troponin or creatine phosphokinase-MB over ≥6 hours with a peak value ≥ twice the upper limit of normal; and ECG changes consistent with ischemia or MI, guided by the Minnesota code [4]. By convention, adjudicators used as the troponin decision threshold the 99^th^ percentile or 10% CV value if clearly identified on the report, or if not clearly identified, twice the lowest listed upper limit of normal (ULN). MIs were adjudicated as definite, probable, or possible, and only definite or probable MI events were included in this study as MI events. The highest and lowest levels of troponin and upper limits of normal were also recorded.

After adjudication, cases were further classified into microsize MI or usual MI based on peak troponin levels. Microsize MI was defined as an adjudicated MI with peak troponin no more than five times the lowest listed ULN, and usual MIs were all other adjudicated MIs. We examined clinical characteristics of individuals and in-hospital, 28-day and 1-year age, sex, and race-adjusted mortality in each of the three ACS groups of no MI, microsize MI and usual MI.

### Statistical analyses

We examined differences in participant characteristics among the three ACS groups, no MI, microsize MI, and usual MI. Available characteristics included sociodemographics (age, sex, race) and medical conditions (hypertension, defined as systolic blood pressure ≥140 mmHg or diastolic blood pressure ≥90 mmHg or treated with antihypertensive medications; diabetes, defined as fasting blood glucose ≥126 mg/dl, random blood glucose ≥200 mg/dl, or being treated with diabetes medications; and a history of heart disease, defined as a self-reported history of MI or coronary intervention, or evidence of MI on ECG at baseline). Physiologic variables included body mass index, low-density lipoprotein cholesterol (LDL-C) levels, high density lipoprotein cholesterol (HDL-C) levels, triglyceride levels, and creatinine levels.

After conducting a descriptive analysis, we constructed a logistic regression model to examine associations with microsize MI relative to usual MI, entering the above clinical characteristics into a multivariable model that included all cases of MI. Mortality for ACS, microsize MI, and usual MI was calculated by dividing the number of deaths in the period among those at risk. Specifically, in-hospital mortality was the number of deaths prior to discharge among those hospitalized; 28-day mortality was the number of deaths at 28 days among those surviving to discharge; and 1-year mortality was the number of deaths at 365 days among those surviving to 28 days. These proportions were adjusted for age, race, and sex and tested for statistically significant differences. All analyses were carried out using SAS version 9.1, Cary, North Carolina.

## Results

### Variations in hospital laboratory troponin reporting

No ULN for troponin was provided in 35 laboratory reports, and these hospitals were excluded, resulting in the inclusion of 649 different hospitals located in 490 US cities in 45 states. The type of troponin was troponin-I in 76% of hospitals and troponin-T in 7%; the type of troponin was not specified in 17% of reports.

Sixteen (2%) hospitals specifically identified either the 99^th^ percentile or the 10% CV level, but only one hospital advised clinicians that the 99^th^ percentile should be used as the decision threshold for defining myocardial necrosis. A single ULN was provided in 48% of hospitals without specification of whether it was the 99^th^ percentile or the 10% CV level. More than one ULN was provided in the remaining hospitals. When more than one threshold was reported, it was often in the context of ranges accompanied by interpretive language (e.g., 0–0.04 “normal”; 0.05-1.5 “indeterminate”; >1.5 “consistent with acute MI”). In 13% of the hospitals, there were four or more such ranges.

The name of the manufacturer of the troponin assay would theoretically permit clinicians to examine the tables available online at the International Federation of Clinical Chemists (IFCC) website for 99^th^ percentile values and 10% CV levels (see Additional file 1). However, the name of the assay was provided in only 15 hospitals, and the IFCC was mentioned in only one report.

Because only a single manufacturer (Roche) distributed troponin-T assays during the study period, the 99^th^ percentile and 10% CV values can be approximated using the IFCC table even when no manufacturer is specified on the laboratory report; the decision threshold during the observation period should have been 0.03 [15,19]. However, in half (n = 38) of troponin-T hospital laboratory reports, 0.1 was provided as the ULN. This higher value corresponded to the assay’s WHO cut-point, which is no longer recommended as a decision threshold.

Additional evidence that some hospital laboratories were providing thresholds well above the recommended decision thresholds came from 56 reports in which the ULN provided was higher than the 10% CV level of any assay reported by the IFCC [15,19]. These results suggest that even among the 48% of labs with a single ULN in our study, there is considerable uncertainty around whether the listed ULN is actually the guideline-concordant decision threshold.

### Clinical implications of microsize MI

Of the 1,029 cases of ACS included in the analysis, 493 were classified as ACS without MI, 183 as microsize MIs, and 353 as usual MIs (Table 1). There were proportionately more black participants in the microsize MI group relative to the usual MI group, more individuals with microsize MI had a history of heart disease, and the mean creatinine was lower in the microsize MI group. Framingham Coronary Heart Disease (CHD) risk scores demonstrated a trend for a graded increase in risk across the groups.

**Table 1:** Baseline characteristics of 1029 patients presenting with acute coronary syndrome (ACS)

	**ACS without MI**	**Microsize MI**	**Usual MI**	***P-value***^***†***^
	Tn* < ULN	**ULN ≤ Tn < 5×ULN**	**Tn ≥ 5×ULN**	
	**(n = 493)**	**(n = 183)**	**(n = 353)**	
Age ≥ 65 years,%	62.3	68.3	66.9	0.73
***Black Race,%***	39.8	***38.3***	***28.6***	***0.03***
Female Gender,%	50.9	37.7	32.3	0.21
Obese,%	49.1	40.4	33.4	0.11
Hypertension,%	81.1	76.0	73.7	0.56
Diabetes,%	36.7	39.9	32.0	0.07
***History of Heart Disease,%***	46.7	***48.6***	***38.8***	***0.03***
Triglyceride ≥200 mg/dl,%	18.5	20.8	20.7	0.99
High Density Lipoprotein Cholesterol ≤40 mg/dl,%	33.5	33.3	41.4	0.07
Low Density Lipoprotein Cholesterol ≥160 mg/dl,%	7.5	5.5	9.9	0.08
***Mean creatinine, mg/dL (SD)***	1.0 (0.4)	***1.0 (0.5)***	***1.2 (1.1)***	***0.03***
Framingham CHD Risk score, Mean (SD)	12.6 (11.3)	14.1 (9.6)	16.5 (12.7)	0.07

*Tn = Troponin, ULN = Upper Limit of Normal, MI = Myocardial Infarction.

^*†*^Chi-square test for categorical variables and student t-test for continuous variables used to test differences between microsize and usual MI groups. See text for definition of medical conditions. Bold italicized characteristics differed statistically between microsize and usual MI groups.

The logistic regression analysis of all cases of MI revealed that black race, history of CHD, and creatinine were independent predictors of microsize MI relative to usual MI (Table 2).

**Table 2:** Multivariable logistic regression results testing associations between having a microsize versus usual MI

	**Crude odds ratios**	**Adjusted odds ratios**	**(95% Confidence interval)**		***P-*value**
***Black vs. White race***	***1.55***	***1.62***	***(1.06,***	***2.48)***	***0.03***
Female vs. Male	1.27	0.94	(0.62,	1.44)	0.78
Obese vs. Not Obese	1.35	1.15	(0.76,	1.73)	0.52
Diabetes vs. No Diabetes	1.41	1.19	(0.78,	1.81)	0.43
***History of heart disease vs. none***	***1.49***	***1.63***	***(1.11,***	***2.39)***	***0.01***
Low density lipoprotein cholesterol ≥160 vs. <160 mg/dL	0.53	0.52	(0.24,	1.10)	0.09
High density lipoprotein cholesterol ≤40 vs. >40 mg/dL	0.71	0.78	(0.52,	1.16)	0.22
***Creatinine, per mg/dL***	***0.76***	***0.69***	***(0.47,***	***1.00)***	***0.05***

For definitions of medical conditions, see text. Bolded italicized results indicate statistical significance.

**Figure 1:**
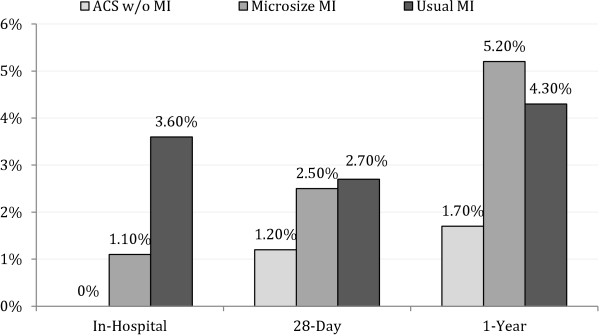
**Adjusted mortality for acute coronary syndrome without MI, microsize MI, and usual MI patients ACS=Acute Coronary Syndrome.** ACS without MI=ACS with peak troponin below upper limit of normal (ULN) and adjudicated as no myocardial infarction (MI). Microsize MI=adjudicated MI with peak troponin above ULN but less than 5 times ULN. Usual MI=adjudicated MI with peak troponin 5 or more times above ULN. All mortality rates include only those surviving the previous period; e.g., 28-day mortality includes only those discharged alive. Total number of deaths: ACS without MI (in-hospital = 0, 28-day = 5, 1-year = 11); Microsize MI (in-hospital = 2, 28-day = 7, 1-year = 12); Usual MI (in-hospital = 7, 28-day = 12, 1-year = 15). ACS w/o MI vs. Microsize MI: In-Hospital p = 0.02 28-Day p = 0.26 1-Year p = 0.02. ACS w/o MI vs. Usual MI: In-Hospital p < 0.01 28-Day p = 0.11 1-Year p = 0.02. Microsize MI vs. Usual MI: In-Hospital p = 0.09 28-Day p = 0.93, 1-Year p = 0.64.

In the mortality analysis, in-hospital mortality was lower for microsize MIs compared with usual MIs, trending toward statistically significant difference (p = 0.09), and in-hospital mortality was statistically different for those with ACS without MI compared to both microsize MI or usual MI (Figure 1). However, although 28-day case fatality was not statistically different across MI groups, mortality trended towards significant differences between the ACS without MI versus the other two groups. At one year, both types of MI had higher mortality relative to those with ACS without MI, and the rate was slightly higher for microsize MIs than for usual MIs, though that difference was not statistically significant.

## Discussion

We found major deviations from guideline recommendations for troponin laboratory reporting in our study. Laboratory reports only rarely identified a 99^th^ percentile or 10% CV level, and more than half of hospitals identified an indeterminate range classifying MI based on a higher threshold than the guideline-recommended approach [1-3,20]. In this context, many microsize MIs may be difficult to recognize clinically.

Although our study was not able to examine the proportion of microsize MIs missed clinically by treating physicians as a result of hospital reporting practices, it is likely that some of these events were in fact not treated as MI. The potential misclassification of microsize MIs as “normal” has significant long-term prognostic implications. The higher long-term mortality risk we observed for those suffering microsize MIs was similar to past reports in which the in-hospital risks were low, but by one month and one year were similar to those with usual MIs [21,22]. This finding underscores both the importance of long-term risk factor management in patients with microsize MIs and the relatively low short-term risks [1-4,21,23-26].

A recent report from Scotland illustrates the clinical importance of detecting microsize MIs [22]. Investigators at the Royal Infirmary in Edinburgh took advantage of the introduction of a more sensitive troponin assay to study clinical management and outcomes among individuals analogous to our microsize MI group. In their study, the focus was on individuals whose peak troponin elevations were above the threshold for the new assay (0.05) and below the threshold for the older, less sensitive assay (0.20), both before and after changes in lab reports. They found that, after introduction of the lower decision threshold on lab reports, secondary preventive measures increased by 15-30% in the microsize MI group but remained similar for those in the usual MI or no MI groups. They also reported reductions in clinical outcomes, including the rate of MIs and deaths at 3 and 12 months in the group with microsize MIs after introduction of the lower threshold on lab reports. While this is an observational study from a single site, it does provide evidence of the potential impact of appropriate troponin reporting to optimize clinical management.

These findings together with our results suggest that improved reporting of hospital troponin results in the US is warranted; the US now has a system of public accountability for the quality of health care that provides a ready infrastructure to enact rapid change. A quality indicator assessing whether the recommended decision threshold is clearly identified on laboratory reports and assessing whether indeterminate ranges are used could be added rapidly to standards for accreditation by the Joint Commission or to the Centers for Medicare and Medicaid Services’ pay-for-performance programs, such as the Premier Hospital Quality Incentive Demonstration [27,28].

Our study included a large proportion of black participants, which has not been the case in previous reports stemming from non-US samples [21]. Our finding that black participants had 62% higher odds for microsize MIs raises concerns that some microsize MIs among black individuals may not be recognized clinically. Indeed, the last decade has demonstrated a concerning widening of the CHD mortality disparity between black and white people in the US, with white mortality rates declining faster than black rates [29]. Similar to past reports, we found that those with a past history of CHD were also more likely to be in the microsize MI group [21]. The clinical consequences of overlooking a microsize MI in individuals with a history of CHD may be minimized because many such patients may already be receiving secondary prevention, including aggressive smoking cessation counseling, lipid management, beta blockers, or anti-platelet therapy. On the other hand, as many as half of individuals on chronic disease preventive therapies discontinue their medications [30-32]; thus, an overlooked microsize MI may translate into a missed opportunity to redouble secondary preventive efforts in these individuals.

Our study’s limitations include our inability to examine how many cases classified as microsize MI by our rigorous adjudication methods were also recognized as MI by the treating physicians. We also did not have available the 99^th^ percentile or 10% CV value at each hospital at the time of the event. Like other large epidemiology studies, we had to rely on clinical laboratories from many hospitals to classify MI and, given the variations we described, it is possible that, while state-of-the art and rigorous, the adjudication process under-detected some microsize MIs. This challenge would bias our results towards finding less difference in mortality rates than may actually exist; thus, the mortality risks we describe may underestimate the true risks. Our team of experts only classified events as MI if there was a clear rising and/or falling pattern of troponin, and perennial low level elevations seen in conditions such as heart failure or renal failure would therefore not have been classified as MI; however, it is possible that some misclassification could have occurred. Unfortunately, this study was not designed to detect differences in clinical management in microsize and usual MIs, but recent reports suggest that this may be an important issue [33]. Future studies should examine what proportion of microsize MIs were not managed as acute MIs. Another limitation is the context of a national epidemiologic cohort study; thus, participants may not be representative of the US population. Although we included 649 hospitals, they may not be representative of all US acute care hospitals. In addition, event accrual may permit future analyses of CHD or cardiovascular disease deaths, in contrast to the all-cause mortality we used in this study.

## Conclusions

Many US hospital laboratories did not report 99^th^ percentiles or 10% CV levels in their troponin reports as recommended by national and international guidelines, complicating the detection of microsize MIs. This may result in misclassification of some individuals with MI, preventing optimal clinical management. Our study confirms the excess risks associated with microsize MI described in other populations, emphasizing the importance of recognizing these cases clinically. Prompt attention to improving the quality of hospital laboratory reporting of patient troponin results is warranted.

## Abbreviations

ACS: Acute coronary syndrome; CHD: Coronary heart disease; CK: Creatine kinase; CV: Coefficient of variation; ECG: Electrocardiogram; HDL-C: High density lipoprotein cholesterol; IFCC: International Federation of Clinical Chemists; LDL-C: Low-density lipoprotein cholesterol; MI: Myocardial infarction; REGARDS: REasons for Geographic And Racial Differences in Stroke; ULN: Upper limit of normal; WHO: World Health Organization

## Competing interests

The authors declare that they have no competing interests.

## Authors’ contributions

MS conceived of the study, participated in its design, adjudicated endpoints, and drafted the manuscript. GP participated in the study design, carried out the analyses and helped to draft the manuscript. CB participated in the study design, collected data, and helped to draft the manuscript. JH adjudicated endpoints and helped with critical redaction of the manuscript. TM conceived of the study, participated in its design, adjudicated endpoints, and critically revised the manuscript. SG adjudicated endpoints and helped with critical redaction of the manuscript. DG conceived of the study, participated in its design and critically redacted the manuscript. RP conceived of the study, participated in its design and critically redacted the manuscript. All authors read and approved the final manuscript.

## Pre-publication history

The pre-publication history for this paper can be accessed here:

http://www.biomedcentral.com/1472-6963/13/162/prepub

## Supplementary Material

Additional file 1 **Commercially available troponin assays obtained from the International Federation of Clinical Chemists, 2006-2008 **^**15,17**^**.** Click here for file
