# Supplementary material for: Hospital laboratory reporting may be a barrier to detection of ‘microsize’ myocardial infarction in the US: an observational study
Source: BMC Health Serv Res. 2013 May 1;13:162. doi: 10.1186/1472-6963-13-162 (PMC3648433; doi:10.1186/1472-6963-13-162)
Supplement: Additional file 1 — Commercially available troponin assays obtained from the International Federation of Clinical Chemists, 2006-2008 15,17. [file 1472-6963-13-162-S1.doc]

| Additional file 1. Commercially available troponin assays obtained from the International Federation of Clinical Chemists, 2006-2008^15,17^ | | | |
| --- | --- | --- | --- |
| **Assay** | **99^th^ Percentile** | | **10% CV** |
| BioMerieux Vidas TnI-Ultra |  | 0.01 | * |
| MKI Pathfast |  | 0.01 | 0.06 |
| Response Biomedical |  | 0.01 | 0.21 |
| Roche Elecsys |  | 0.01 | 0.03 |
| Roche E170 |  | 0.01 | 0.03 |
| Abbott ARCH |  | 0.012 | 0.032 |
| Innotrac Aio! |  | 0.023 | 0.036 |
| Mitsubishi chemical |  | 0.029 | * |
| Ortho ES (R&D) |  | 0.032 | 0.053 |
| Ortho Vitros Eci |  | 0.034 | 0.034 |
| Abbott AxSYM ADV |  | 0.04 | 0.16 |
| Bayer Ultra |  | 0.04 | 0.03 |
| Beckman Access AccuTnI |  | 0.04 | 0.06 |
| Siemens Centaur RnI-Ultra |  | 0.04 | 0.03 |
| Siemens VISTA |  | 0.045 | 0.04 |
| Inverness Biosite Triage |  | 0.05 | * |
| Roche Reader |  | 0.05 | * |
| Tosoh AIA |  | 0.06 | 0.09 |
| Dade RxL |  | 0.07 | 0.14 |
| Dade CS |  | 0.07 | 0.06 |
| Siemens Dimension RxL |  | 0.07 | 0.14 |
| Siemens Stratus CS |  | 0.07 | 0.06 |
| Abbott i-STAT |  | 0.08 | 0.1 |
| Bayer Centaur |  | 0.1 | 0.35 |
| DPC Immulite |  | 0.2 | 0.6 |
| Siemens Immulite 2500 STAT |  | 0.2 | 0.42 |
| Siemens Immulite 1000 Turbo |  | * | 0.64 |

*Not available
